# A Role for MRE11, NBS1, and Recombination Junctions in Replication and Stable Maintenance of EBV Episomes

**DOI:** 10.1371/journal.pone.0001257

**Published:** 2007-12-05

**Authors:** Jayaraju Dheekollu, Zhong Deng, Andreas Wiedmer, Matthew D. Weitzman, Paul M. Lieberman

**Affiliations:** 1 The Wistar Institute, Philadelphia, Pennsylvania, United States of America; 2 The Salk Institute, La Jolla, California, United States of America; Duke University, United States of America

## Abstract

Recombination-like structures formed at origins of DNA replication may contribute to replication fidelity, sister chromatid cohesion, chromosome segregation, and overall genome stability. The Epstein-Barr Virus (EBV) origin of plasmid replication (OriP) provides episomal genome stability through a poorly understood mechanism. We show here that recombinational repair proteins MRE11 and NBS1 are recruited to the Dyad Symmetry (DS) region of OriP in a TRF2- and cell cycle-dependent manner. Depletion of MRE11 or NBS1 by siRNA inhibits OriP replication and destabilized viral episomes. OriP plasmid maintenance was defective in MRE11 and NBS1 hypomorphic fibroblast cell lines and only integrated, non-episomal forms of EBV were detected in a lympoblastoid cell line derived from an NBS1-mutated individual. Two-dimensional agarose gel analysis of OriP DNA revealed that recombination-like structures resembling Holliday-junctions form at OriP in mid S phase. MRE11 and NBS1 association with DS coincided with replication fork pausing and origin activation, which preceded the formation of recombination structures. We propose that NBS1 and MRE11 promote replication-associated recombination junctions essential for EBV episomal maintenance and genome stability.

## Introduction

Genome stability depends on the successful completion of chromosome duplication prior to cell division [1]. Damaged DNA, nucleotide depletion, and endogenous nucleoprotein structures may present significant challenges to replication fork progression and prevent the completion of DNA synthesis. Several mechanisms are known to overcome these challenges, including the utilization of multiple origins of DNA replication and an elaborate post-replication repair system [2]. One important source of post-replication repair involves the use of homologous recombination between sister chromatids [3]. Homologous recombination-based mechanisms have been implicated in replication re-initiation at sites of collapsed replication forks and double strand breaks. Proteins implicated in homologous recombination are known to play a role in replication fork formation, stability, and processing [1], [4]–[6]. Many of these homologous recombination repair proteins, including MRE11, NBS1, RAD50, BRCA1, and BRCA2 for example, are commonly mutated in human cancers and heritable genome instability disorders [5]–[7]. However, the precise function of these homologous recombination repair proteins during normal DNA replication are only beginning to be understood in molecular detail.

Epstein-Barr Virus (EBV) provides a unique, and clinically significant model system to study genome stability in human cells. EBV is a gamma herpesvirus that establishes a long-term stable episomal infection in B-lymphocytes [8]. The latent infection is tightly linked to several human cancers, including forms of Burkitt's lymphoma, Hodgkin's disease, and nasopharyngeal carcinomas [9], [10]. The viral episome replicates synchronously with the cellular genome and utilizes many of the same replication and cell cycle licensing factors as chromosomal DNA [11]–[16]. The plasmid stability of EBV is conferred by a small genetic element, referred to as OriP, which consists of a family of repeats (FR) and a dyad symmetry (DS) element [17], [18]. The repeats in the FR bind to the viral encoded nuclear antigen, EBNA1, which is essential for viral maintenance and immortalization of primary lymphocytes [19], [20]. The DS region consists of phased EBNA1 sites juxtaposed with telomere repeat factor (TRF) binding sites, which together function as an efficient origin of DNA replication initiation [21]–[23] . However, the DS must function coordinately with the FR for stable episomal maintenance [24].

TRF2 binding sites within DS contribute to the DNA replication activity and plasmid stability of OriP-containing plasmids [21], [22], [25]–[27]. One function of TRF2 at DS is to facilitate the recruitment of cellular origin recognition complex (ORC) [28]. At cellular telomeres, TRF2 is essential for the protection of chromosome ends from double-strand break repair and end-to-end fusions [29], [30]. TRF2 stabilizes the lariat-like structure at the end of telomeres, referred to as the T-loop, which is formed by the single strand 5′-end invasion of the duplex DNA within the telomere repeats [31], [32]. The precise role of TRF2 in telomere repeat DNA strand invasion and DNA replication is not completely understood, but it has been proposed that TRF2 regulates branch migration and may bind to recombination-like structures resembling Holliday junctions [33], [34].

Homologous recombination proteins interact with telomere repeat binding factors and contribute to telomere DNA replication and maintenance [35]. TRF2 can be isolated in a complex with components of the MRE11/RAD50/NBS1 (MRN) complex and the association with NBS1 was found to be cell cycle regulated [36]. The MRN complex is a highly conserved heterotrimer that functions in several different aspects of cellular DNA repair, recombination, and checkpoint surveillance [6]. MRE11 has both endo- and exonuclease activity, while RAD50 forms a zinc-hook capable of linking neighboring molecules [37]. The MRN complex accumulates at double strand break-induced foci, along with several other cellular proteins, including 53BP1, CHK2, and BRCA1. In addition to their role at sites of damaged DNA, MRN and other members of the intra-S phase checkpoint response have been implicated in normal replication initiation events at origins and during re-initiation at sites of stalled or collapsed replication forks [38].

In earlier studies, MRE11 and NBS1 have been implicated in the replication activity of OriP through an association with the E2F binding sites located over 1 kb from the DS region [39]. We now provide evidence that MRE11 and NBS1 can be recruited to the DS region of OriP through additional interactions, including those with TRF2, and that deficiencies in MRE11 and NBS1 inhibit Orip replication and maintenance function. We present evidence that that recombination-like structures form in the later stages of S phase at OriP. We suggest that these recombination structures form as a consequence of the pausing and initiation, as has been proposed for origins of replication in lower eukaryotes [40]–[42]. We also suggest that recombination structures formed at OriP provide a junction between sister chromatids that are important for cohesion and segregation of EBV episomes.

## Results

### Telomere repeats enhance MRE11 binding to OriP

**Figure 1:**
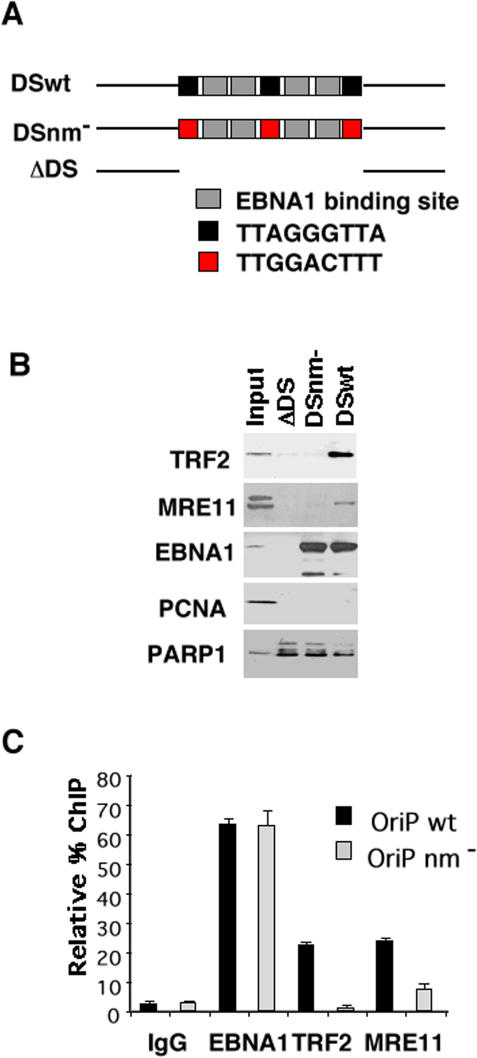
MRE11 associates with the DS region of OriP. A) DNA affinity purification assays were performed with Raji nuclear extracts and DNA templates derived from DS wt, DS nm-, or ΔDS. Input and template bound proteins were analyzed by Western blot with antibodies to TRF2, MRE11, EBNA1, PCNA, and PARP1, as indicated. B) Schematic of template DNA used for DNA affinity purification in A, and their respective protein binding sites. C) ChIP assays to monitor EBNA1, TRF2, and MRE11 binding at OriP wt or OriP nm- in transfected 293 cells. Immunoprecipitated DNA was quantified by real time PCR relative to total input DNA recovered for each transfected cell.

Previous work has shown that TRF2 binds to the three telomere repeats within the DS region of OriP [21], [22]. Since TRF2 can interact with the MRN components, we tested whether MRE11 could be recruited to DS in vitro using a DNA affinity purification assay (Fig. 1A). We compared DNA templates containing wild type DS (DSwt), DS with substitution mutations in the telomere repeats (DSnm-), or OriP DNA with a 120 bp deletion of the DS (ΔDS) (Fig. 1B). As expected, TRF2 bound efficiently to DS wt, but not to DS nm- or ΔDS. Noteably, MRE11 was detected in the DSwt, but not in any of the control templates lacking telomere repeats. Control protein EBNA1 bound to DS wt and DS nm-, but not to ΔDS lacking EBNA1 binding sites. Replication protein PCNA did not bind to any of the templates, and the DNA binding protein PARP1 bound non-specifically to all of the templates. These findings indicate that MRE11 can be recruited to DS template DNA in vitro, and that binding depends on the telomere repeat sequence in DS.

To determine if MRE11 bound to DS in vivo, we used the chromatin immunoprecipitation (ChIP) assay followed by real time PCR (Fig. 1C). We tested the effect of the telomere repeats on protein interactions by comparing OriP wt and OriP nm- containing plasmid in transfected cells. We found that EBNA1 bound to OriP wt and nm- to a similar extent. TRF2 bound OriP wt (∼22% input), but its binding was reduced over 10 fold (∼1.5% input) for OriP nm-. We also found that MRE11 bound OriP wt (∼26% input), but was reduced by 4 fold (∼6.5% input) for OriP nm-. These findings indicate that MRE11 binds to OriP in vivo, and that telomere repeats contribute to this binding.

### MRE11 and NBS1 contribute to OriP replication and episome maintenance function

**Figure 2:**
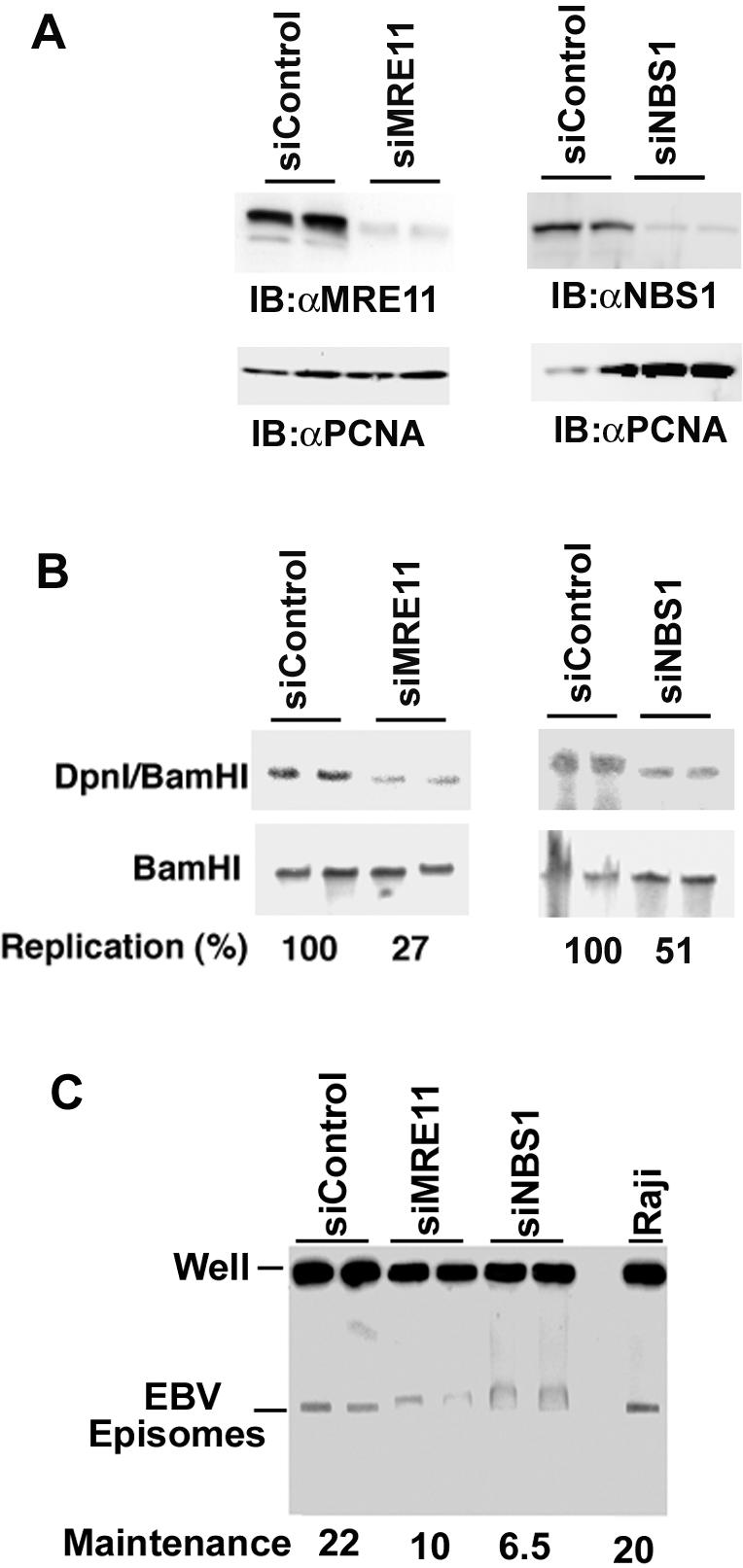
MRE11 and NBS1 contribute to OriP replication and EBV episome stability. A) D98/HR1 cells were transfected with siRNA for MRE11 or NBS1, or Luciferase control and assayed by immuno-blot (IB) with antibodies specific for MRE11 (left pane) or NBS1 (right panel), or PCNA (lower panel) as a loading control. B) Transient DNA replication assays of OriP-containing plasmids were analyzed by Southern blot. D98/HR1 cells were transfected with OriP plasmid and siRNA for either MRE11, NBS1, or luciferase control (as indicated above each lane). Hirt extracted plasmid DNA was digested with DpnI plus BamHI (top panel) or BamHI only (lower panel) and probed with OriP specific probes. DNA was quantified by PhosphorImager analysis and the replication value was determined as the ratio of DpnI/BamHI to BamHI recovered products. Quantification shown below is a summary of at least four independent replication assays (data not shown). C)) EBV episomes from latently infected D98/HR1 cells were analyzed by pulse field electrophoresis and Southern blotting after transfection of siRNA for control (siLuc), MRE11, or NBS1. Raji cells were used as a control for EBV episome size and abundance. The percentage of EBV episomes relative to the EBV DNA retained in the well is calculated below.

To determine if MRE11 and NBS1 contribute to OriP replication functions, we assayed the effects of siRNA depletion on OriP plasmid replication (Fig. 2). For these experiments, we used the EBV positive adherent cell line D98/HR1 which carry EBV episomes and have relatively high transfection efficiency. siRNA depletion of MRE11 and NBS1 was estimated at greater than 80% reduction as monitored by Western blotting (Fig. 2A). OriP-dependent plasmid replication was assayed by Southern blot analysis of Dpn I resistant plasmid (top panel) relative to total input DNA linearized by BamHI (lower panel) (Fig. 2B). The DpnI resistant form measures the DNA replicated in mammalian cells since its methylation pattern differs from the E. coli generated plasmid DNA initially transfected. PhosphorImager quantification of at least four independent transfections revealed that siRNA depletion of MRE11 and NBS1 reduced OriP replication by ∼3.7 and ∼2.0 fold, respectively (Fig. 2B, and data not shown). MRE11 and NBS1 depletion had no appreciable effect on D98/HR1 cell proliferation nor on cell cycle distribution as measured by propidium iodide staining and FACS analysis of transfected cells (Fig. S1). This suggests that the MRE11and NBS1 depletion has a modest, but consistent inhibitory effect on OriP replication.

OriP is also essential for episome maintenance of EBV. To determine if MRE11 or NBS1 contribute to EBV episome maintenance, we examined the stability of EBV episomes in D98/HR1 cells after siRNA depletion of MRE11 and NBS1. The episomal form can be detected by pulse field gel (PFG) electrophoresis and Southern blotting of D98/HR1 cell DNA purified in agarose plugs (Fig. 2C). We found ∼22% of the EBV DNA could be isolated as intact episomes in D98/HR1 cells transfected with control siRNA (siControl). The DNA retained in the well is a combination of integrated and trapped episomal DNA. This was similar to that observed for latently infected Raji cells, which are known to have mostly episomal, but some integrated forms of EBV DNA. Transfection of D98/HR1 cells with siRNA specific for MRE11 or NBS1 decreased EBV episomes to ∼10 and 6.5% of total EBV DNA, corresponding to a 2.2 and 3.1 fold loss of episomes in five days (Fig. 2C). MRE11 and NBS1 depletion also caused aberrant migration of EBV episomes in PFG electrophoresis, suggesting that these proteins contribute to the structural stability of latent EBV genomes. These effects suggest that MRE11 and NBS1 function in EBV episome maintenance, as well as in genome stability.

**Figure 3:**
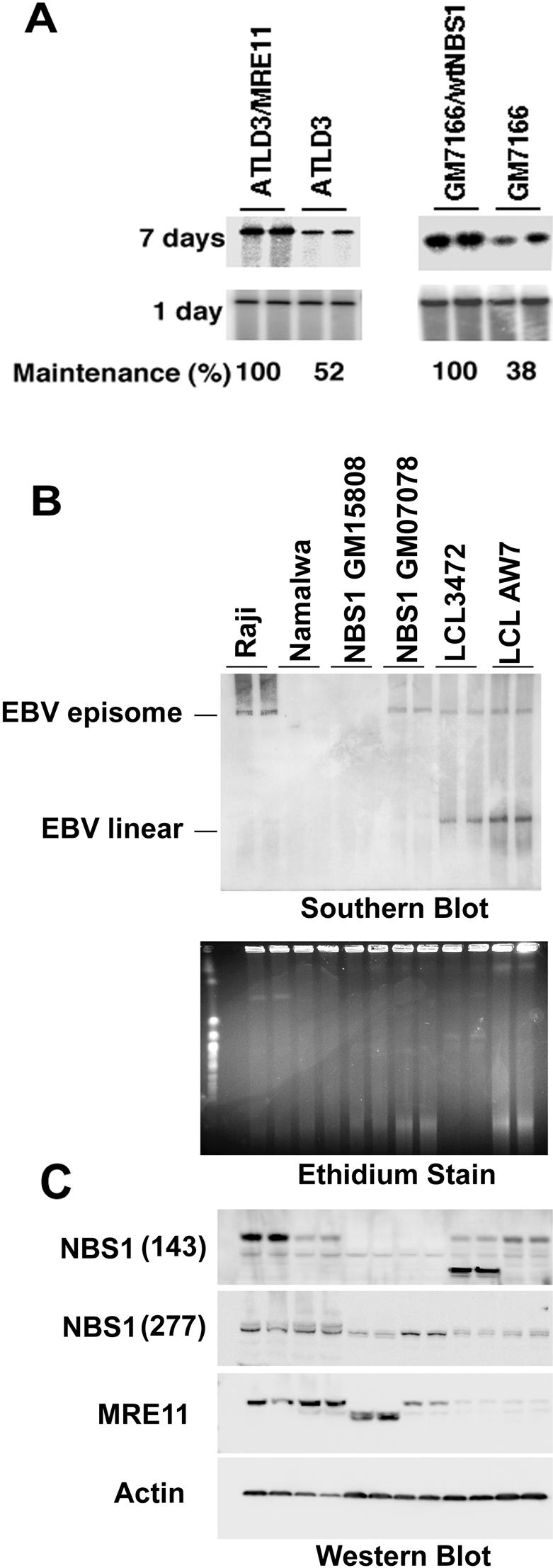
Evidence that MRE11 and NBS1 are required for EBV episome maintenance. A) OriP plasmid maintenance assays were performed in MRE11 mutant (ATLD3) and reconstituted (ATLD3/wtMRE11) cell lines (left panel) or NBS1 mutant (GM7166) and reconstituted (GM7166/wtNBS1) cell lines (right panel). Plasmids containing OriP and EBNA1 were monitored by Southern blotting of Hirt lysates at 1 (lower panel) and 7 (upper panel) days post-transfection. Phosphorimager quantification of four independent experiments as shown in panal A where maintenance is measured as the ratio of day 7 to day 1 for OriP plasmid detection is shown below. B) EBV transformed B-lymphocytes were analyzed by PFG and Southern blotting for the presence of episomal forms of the EBV genome. Raji, Namalwa, NBS1 (GM15808), NBS1 (GM07078), LCL3472, and LCLAW7 DNA was loaded at equal concentrations and analyzed by ethidium staining of PFG (lower panel) or by Southern blot (upper panel) with EBV specific probe. C) Western blot of the same cell lines used for PFG analysis shown in panel B was probed with antibodies specific for NBS1 (antibody 143 and antibody 277), MRE11, or loading control Actin.

To further assess the role of MRE11 and NBS1 in OriP function, we assayed OriP plasmid maintenance in MRE11 and NBS1 hypomorphic cell lines. Isogenic cell lines derived from mutant and wt replacements of MRE11 and NBS1 have been described previously [43]. Cell lines mutated in MRE11 (ATLD3) or NBS1 (GM7166) and there corresponding wild-type corrected cell lines ATLD3/wtMRE11 or GM7166/wtNBS1, respectively, were tested for their ability to support OriP plasmid maintenance (Fig. 3A). Wt and mt cell lines were transfected with OriP plasmid expressing the EBNA1 protein, and then assayed for plasmid maintenance at 1 day or 7 days post-transfection. We found that MRE11 and NBS1 mt cell lines were impaired for maintaining OriP plasmids after 7 days post-transfection, with no detectable difference observed at 1 day post-transfection (Fig. 3A). Wt and mt cell lines proliferated at similar rates, with similar cell cycle profiles (data not shown), and identical cell numbers were analyzed for each cell type. Quantification of the average of four independent experiments revealed that MRE11 and NBS1 mutant cell lines were reduced by ∼2 and ∼2.5 fold for plasmid maintenance relative to wt cell lines.

One prediction of these studies is that MRE11 and NBS1 are required for stable episome maintenance of EBV during lymphoblastoid cell transformation. To test this prediction, we analyzed the structure of EBV genomes in two lymphoblastoid cells derived from NBS1 mutated patients (Fig. 3B). EBV episomes were monitored by Southern blotting of agarose gels after PFG electrophoresis. Episomes were readily detected in Raji cells, which are known to carry multiple copies of circularized EBV genomes. In contrast, no episomes were detected for Namalwa Burkitt lymphoma cellls, which are known to have integrated EBV genomes and lack episomal forms [44]. Interestingly, we found that NBS1 GM15808 cells had no detectable episomes (Fig. 3B). More extensive analysis of the EBV termini indicated that GM15808 cells had integrated viral genomes, similar to Namalwa (Fig. S2). In contrast, a second NBS1 mutated cell line GM0708 had EBV episomes at levels comparable to that of other lymphoblastoid cell lines, LCL3472 and LCLAW7. Simlar preparations of total cellular DNA were analyzed for each sample as demonstated by ethidium staining of the PFG (Fig. 3B, lower panel). To determine if the lack of episomes in NBS1 GM15808 correlates with NBS1 and MRE11 protein expression or structure, we analyzed these cell lines by Western blot analysis (Fig. 3C). We found that NBS1 protein was absent from both NBS1 cell lines when assayed with antibody 143. In contrast, we found that NBS1 protein was detectable, but with a slightly faster mobility when probed with antibody 277, which recognizes a different NBS1 epitope. Interestingly, we found that MRE11 protein had a markedly faster mobility in NBS1 GM15808 cells relative to all other cell types analyzed, including NBS1 GM07078 (Fig. 3C). Since lymphoblastoid cells are non-clonal expansions of EBV immortalized B-lymphocytes they are expected to have episomal EBV. The lack of episomes in GM15808 suggest that their more severe defect in both NBS1 and MRE11contributes to their failure to support episomal maintenance of EBV.

### Cell cycle-dependent recruitment of MRE11 and NBS1 to episomal OriP

**Figure 4:**
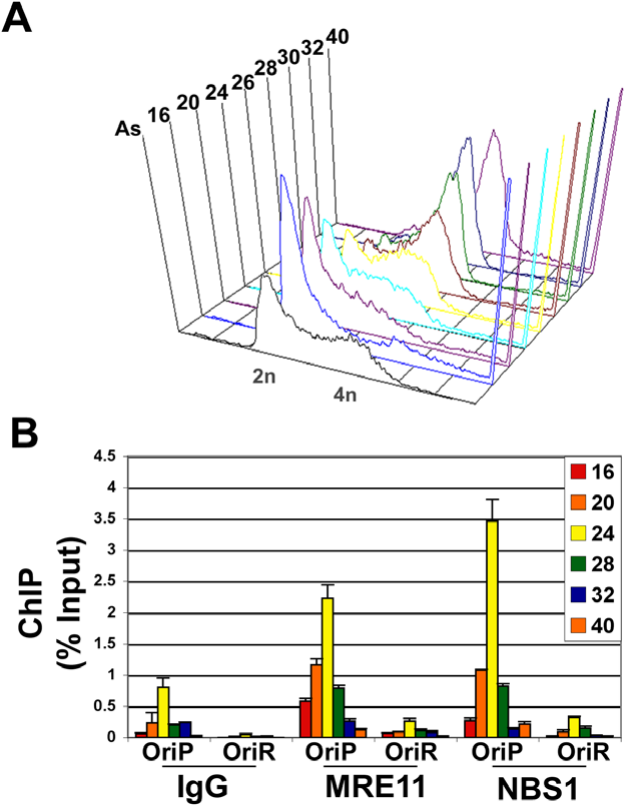
Early S phase recruitment of MRE11 and NBS1 to DS. A) Mutu I cells were fractionated by centrifugal elutriation and analyzed for cell cycle synchronization by FACS. Cell cycle fraction numbers correspond to rates of counter flow used for centrifugal elutriation. B) MutuI cells were fractionated by centrifugal elutriation as shown in panel B, and then analyzed by chromatin immunoprecipitation with antibodies to MRE11, NBS1, and control IgG. Immunoprecipitated DNA was quantified by real-time PCR with primers specific for OriP or control OriR regions of the EBV genome. ChIP values were presented as percentage of input DNA. Cell cycle fractions correspond to the FACS analysis in panel A.

MRN is known to have cell cycle-specific activities, and interact with TRF2 in a cell cycle-dependent manner. We therefore examined whether MRN components, MRE11 and NBS1 associate with OriP in a cell cycle-dependent manner (Fig. 4). MutuI Burkitt lymphoma cell lines maintain multiple copies of the latent EBV episome. MutuI cells were fractionated by centrifugal elutriation to isolated cell cycle synchronized populations. Cell cycle synchronizations were verified by FACS analysis after propidium iodide staining (Fig. 4A). Cells isolated at different stages of the cell cycle were assayed by ChIP for binding of MRE11 and NBS1. We compared binding to OriP with a control region within the alternative replication initiation zone used in Raji cells, referred to as OriR [45]. ChIP values were quantified as percentage of input DNA for different stages of the cell cycle and for each region of the genome. We found that MRE11 and NBS1 were enriched at OriP, but not at OriR. We found a significant cell cycle-dependent association of MRE11 and NBS1 at OriP, with ∼10 fold increase in binding in mid-S phase (fraction 24) relative to early G1 (Fig. 4B). MRE11 and NBS1 were also recruited to OriP in mid S phase when cells were synchronized by double thymidine block and release (Fig. S3), indicating that the method of synchronization was not responsible for the interaction or timing. Furthermore, high levels of DNA damaging agents, including gamma-irradiation (IR) or 5 mM hydroxyurea (HU), caused a depletion of MRE11 and NBS1 from OriP (Fig. S3B). These findings suggest that cell cycle association of MRE11 and NBS1 with OriP is distinct from the general cellular response to DNA damage induced by HU and IR.

### Recombination-like structures form at OriP

**Figure 5:**
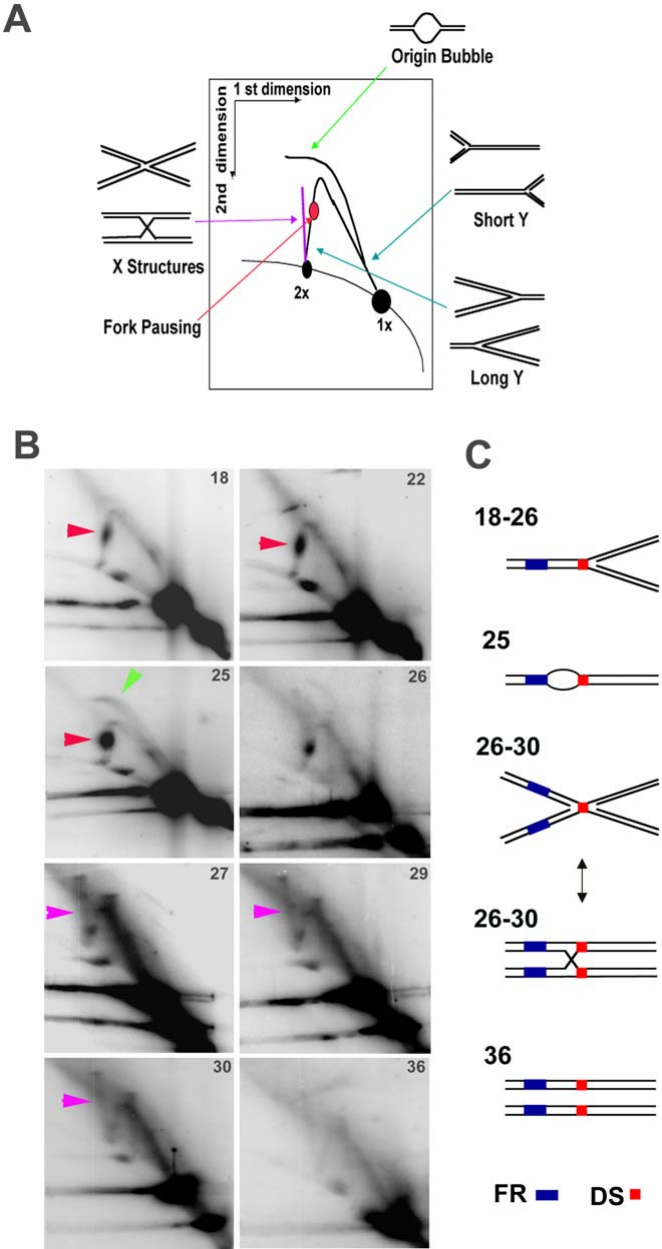
Cell cycle analysis of replication and recombination structures formed at OriP DNA. A) Schematic of replication and recombination-associated DNA structures resolved by 2-dimensional neutral agarose gels. Holliday junction and X-like structures migrate as a vertical spike emerging from the 2n spot (shown in magenta). The bubble arc (shown in green) and replication fork pause sites (shown in red) are also indicated. B) Two dimensional neutral agarose gel analysis of OriP isolated using CTAB method from Mutu I cells after cell cycle fractionation. Cell cycle fractions from centrifugal elutration are indicated in the upper right corner of each image. C) Cartoon interpretation of the major OriP DNA structures observed for each cell cycle fraction in panel C.

MRE11 and NBS1 have been implicated in the homologous recombination pathway and intra-S phase checkpoint response. Since MRE11 and NBS1 localize to OriP in mid-S phase, we explored the possibility that recombination-like structures may form at OriP. The DNA structures formed during replication and recombination can be analyzed using two-dimensional neutral agarose gel (2D gel) electrophoresis. The migration properties of prominent DNA structures have been well established, including the Y-arcs formed by replication fork progression, replication pause sites that appear as bulges in the Y-arc (Fig. 5A, red arrows), origin bubbles arcs (Fig. 5A, green arrow), and Holliday junctions or X-structures which appear as vertical spikes emerging from the 2n spot (Fig. 5A, magenta arrows). Holliday junctions are notoriously unstable due to their transient nature and branch migration on linearized fragments. To enrich for recombination-like structures, DNA was isolated with the cationic-detergent CTAB, which inhibits branch migration during DNA isolation and manipulation [46]. To further enrich for transient recombination structures, and to determine if recombination structures formed at OriP at times when MRE11 and NBS1 associated with OriP, we analyzed cells that were isolated from different stages across the cell cycle using centrifugal elutriation, as shown in Fig. 4A. Cell cycle fractions from EBV positive Mutu I cells were analyzed by 2-D gel electrophoresis (Fig. 5B). At early stages of S phase, we found accumulation of Y-structures and the formation of a pronounced fork pausing site (Fig. 5C, red arrows, fractions 18, 22, 25). This pause was most likely localized to the DS region based on the appearance in the large Y-arc of the PvuII digested OriP DNA. Further progression through S phase revealed that the formation of a bubble arc indicative of replication initiation (green arrow, fraction 25). As cells further progress through mid S phase, the replication pause sight disappeared, and a vertical spike emerging from the 2n position appeared. This vertical spike has been interpreted as recombinational intermediates, which may include Holliday junctions, X-structures, and hemicatenanes (magenta arrows, fractions 27, 29, 30). By G2/M, the major replication and recombination structures were less apparent (fraction 36). Fork pausing and recombination-like structures were also observed at OriP in Raji cells (Fig. S4). A schematic representation of these structures is presented in Figure 5C.

**Figure 6:**
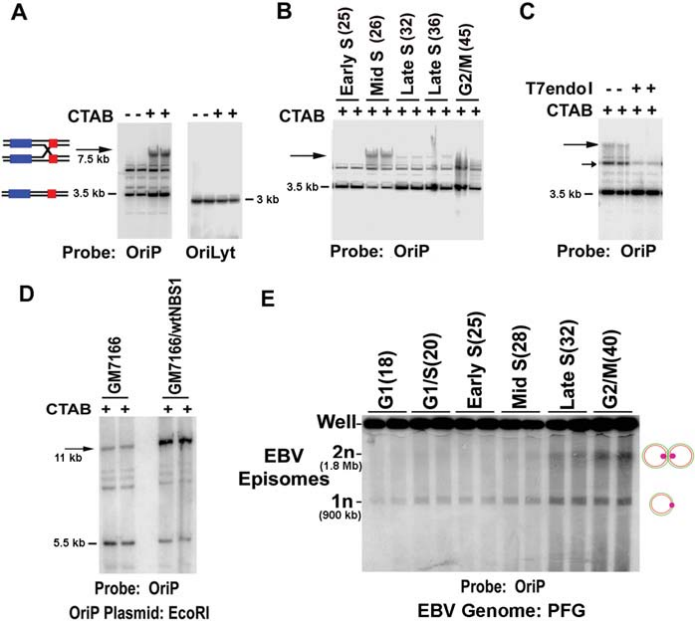
Evidence for recombination junctions formed at OriP. A) MutuI cell DNA was extracted with (+) or without (−) CTAB to stabilize recombinational structures. DNA was isolated and cleaved by EarI and then analyzed by Southern blot from a one-dimensional agarose gel with probes to OriP (left panel) or OriLyt (right panel). The position of the expected 1n products are indicated. B) MutuI cells were fractionated by centrifugal elutriation as shown for Fig. 4B. DNA was extracted using CTAB method and cleaved with EarI, and analyzed by Southern blot with probe to OriP. C) DNA was extracted from MutuI cells with CTAB, digested with EarI, and then mock treated (lanes 1 and 2), or treated with T7 endonuclease I (lanes 3 and 4) or with mung bean nuclease (lanes 5 and 6). DNA fragments were visualized by Southern blot with probe to OriP. D) NBS1 mt (GM7166) or reconstituted (GM7166/wtNBS1) cell lines were transfected with plasmid OriP and selected for 48 hrs using hygromycin. Total cell DNA was isolated using CTAB method, linearized with EcoRI, and then detected by Southern blot with probe specific for plasmid OriP. The 5.5 kB linear DNA fragment (1n) and slower mobility form (arrow) are indicated. E) Pulse field electrophoresis and Southern blot of EBV episomes isolated from MutuI cells at different stages of the cell cycle, as indicated above each pair of lanes. The 1n position corresponds to a monomeric circular genome (∼900 Kb of linear DNA), while the 2n corresponds to a dimeric circular genome (∼1.8 Mb of linear DNA).

Two-dimensional gel electrophoresis of CTAB isolated DNA provided evidence that recombination-like structures form at OriP (Fig. 5). These structures were further analyzed using one dimensional gel electrophoresis (Fig. 6). DNA was extracted from MutuI cells with or without CTAB, digested with EarI restriction, and then analyzed by Southern blotting (Fig. 6A). Analysis with OriP specific probe revealed the predicted ∼3.4 kb (1n) species, as well as several slower migrating forms. When DNA was extracted with CTAB, we observed a novel OriP containing species migrating at ∼7.5 kb. Reprobing the same blot for a ∼3.2 kb fragment adjacent to EBV OriLyt showed no structures dependent on CTAB extraction. This indicates that a CTAB-stabilized structure migrating as ∼7.5 kb occurs at OriP, but not at OriLyt-containing DNA.

Our 2D gel analysis (Fig. 5) suggested that recombination-like structures form in mid to late S phase (fractions 26–30). We therefore assayed the formation of 2n species across the cell cycle using MutuI cells that were fractionated by centrifugal elutriation (Fig. 6B). We found that the 7.5 kb CTAB-stabilized species (indicated by the arrow) formed predominantly in mid S phase (fraction 26). This species was not detected in cells derived from early S phase (fraction 25), or G2/M (fraction 45), and was detectable in small amounts during late S phase (fractions 32 and 36). The appearance of the 7.5 kb structure coincides with the formation of the spike structure in 2D gels (Fig. 5).

The DNA structure formed at OriP was further investigated with the use of a structure-specific endonuclease. Holliday junctions (HJ) and related recombinational structures are known to be cleaved by T7 endonuclease I (T7endoI) [47]. MutuI cell DNA was extracted with CTAB and linearized with a EarI as above. The restricted DNA was then subject to digestion with or without T7endoI and assayed by one-dimensional gel and Southern blotting with OriP-specific probe (Fig. 6C). We found that T7endoI eliminated the 7.5 kb CTAB-sensitive species. T7endoI also partially digested a faster migrating 6.0 kb (small arrow) species, with a corresponding increase in the faster migrating (1n) species. We also found that T7endoI eliminates the vertical spikes, along with replication pause sites, observed in 2D neutral agarose gels (Figs. S5 and S6). Additionally, incubation at 65°C which facilitates branch migration and the resolution of HJ structures on linear DNA, reduced the appearance of the vertical spikes in 2D gels (Fig. S6). We conclude that the vertical spike observed in 2D gels, and the CTAB-stabilized structures observed in 1D gels, are consistent with HJ and related recombination structures.

We next asked whether the MRN complex contributed to the formation of the slower migrating forms of OriP DNA. NBS1 hypomorphic cell line GM7166, or NBS1-wt reconstituted cells GM7166/NBS1wt were transfected with OriP plasmid and selected for 48 hrs prior to extraction with CTAB and linearized with EcoRI restriction endonuclease (Fig. 6D). In GM7166/NBSwt cells, we observed a slower migrating species (∼11 kb, indicated by the arrow) that formed 6 fold greater relative to the 1n species (∼5.5 kb EcoRI fragment). In GM7166 cells, the slower migrating species formed only ∼1.6 fold over the 1n species (Fig. 6D). These results indicate that NBS1 contributes to the formation of the slower migrating form of OriP DNA, and suggests that NBS1 is required for the formation of recombinational structures at OriP.

The recombination junctions that form at OriP are predicted to form linkages between newly replicated sister EBV genomes. To test this possibility, we analyzed EBV genome migration by PFG electrophoresis at different stages of the cell cycle (Fig. 6E). Cell cycle fractions were collected by centrifugal elutriation as described in Fig. 4A and EBV episomes were analyzed by PFG electrophoresis and Southern blotting as described in Fig. 2C and 3B. We found that monomeric episomes were the predominant form in G1 and early S, migrating at a position equivalent to ∼900 kb of linear DNA . A slower migrating species with the predicted mobility of a 2n dimeric circle (∼1.8 MB of linear DNA) appeared in mid S and accumulated to almost 50% of the total molecules by G2/M (Fig. 6E). This finding is consistent with the model that EBV genomes form topologically linked 2n species after DNA replication.

## Discussion

Intra-S phase checkpoint proteins are thought to monitor and process replication intermediates formed during DNA damage conditions, as well as during normal cellular division [48], [49]. The MRN complex, in particular, is known to colocalize with sites of DNA replication and is thought to stabilize and repair stalled or collapsed replication forks [6], [39], [50]. MRN components have also been implicated in the replication and genome stability of several DNA viruses, including herpes simplex [51]. However, the precise role of the MRN in cellular DNA replication processing remains elusive, partially as a consequence of having limited knowledge of the genetic loci where MRN functions. Here, we provide evidence that components of the MRN complex function at the replication origin and plasmid maintenance element of Epstein-Barr virus. We provide evidence that recombination-like X structures form at OriP following replication pausing and origin activation. We suggest that MRN proteins regulate the formation and/or processing of origin-associated recombinational structures, and that these structures contribute to the plasmid maintenance function of OriP.

### Cell cycle-dependent association and function of MRN at DS

Telomere repeats within DS contribute to the DNA replication and plasmid maintenance function of OriP [21]. It has been previously shown that TRF2 associates with components of the MRN complex [36], [52]. We therefore explored the potential role of MRE11 and NBS1 in the functions of OriP. We found that MRE11 associates with the DS region of OriP and that the telomere repeats enhanced this association (Fig. 1). We also found that MRE11 and NBS1 bound to DS in a cell cycle-dependent manner with maximum binding occuring in middle S phase (Fig. 4). A cell cycle-dependent interaction of NBS1 with TRF2 has been previously reported, thus potentially explaining the cell cycle association of MRN with the DS region of OriP [36]. Deletion of telomere repeat sites in DS only partially eliminated MRN binding in vivo, suggesting that other mechanisms also contribute to MRN recruitment to OriP. An E2F site located ∼1 kb from DS can also contribute to the localization of MRN at OriP [39]. However, it is also possible that MRN is recruited to DS through the recognition of complex DNA structures associated with replication initiation, pausing, and termination. We speculate that TRF2 sites evolved at DS to reinforce MRN recruitment and enhance the processing of DNA structures important for episome maintenance.

The function of MRN proteins at DS was investigated using the DNA replication and plasmid maintenance assays. We found that OriP replication activity and EBV genome stability was reduced in cells where MRE11 and NBS1 were depleted by siRNA (Fig. 2). We also found fibroblasts with hypomorphic mutations in MRE11 or NBS1 were defective in maintaining OriP-dependent plasmids (Fig. 3). Furthermore, an EBV immortalized lymphoblastoid cell line derived from an NBS1 mutated individual lacked any detectable episomal forms of EBV (Fig. 3B). Although a second NBS1 mutated lymphoblastoid cell line retained EBV episomes, this second cell line had normal MRE11 protein and less severe alterations in NBS1 (Fig. 3C). Since the EBV transformed NBS1 cells are not a clonal isolate, we consider the exclusively integrated form to be highly significant, since it represents the statistical average of many transformation events. Almost all non-cloned LCLs have episomal and linear forms of EBV. The NBS1 mutated cells had no linear forms, and in the case of the more severely mutated NBS1 and MRE11 defective cell line GM15808, there were no detectable episomes. These findings argue strongly that NBS1 and MRE11 are important for maintaining stable episomes of EBV during latent infection in lymphoblastoid cells. Since MRE11 and NBS1 localize to DS by ChIP, and not to other regions of the OriP plasmid, we consider it likely that MRN acts directly at DS to process DNA structures formed during DNA replication that are necessary for stable episome maintenance.

### Replication pausing, initiation, and termination at OriP

Two dimensional agarose gel analysis of OriP has revealed that replication can both initiate, pause and terminate near the DS and FR regions of OriP [53]–[56]. Our data shows that a replication pause site forms within or adjacent to DS early in S phase (Fig. 5B, factions 18), prior to origin bubble arc formation (Fig. 5B, fraction 25), and persists until mid S phase (factions 29). Weaker pause sites could also be detected at FR. This suggests that EBNA1 sites at DS and FR obstruct replication fork progression. The accumulation of Y-structures at OriP is most probably a consequence of replication initiating outside of OriP. Single molecule analysis of DNA replication revealed that replication can initiate at other sites in EBV, and that OriP is a major site of replication fork pausing and termination [57]. In this scenario, the origin function of DS may be required only under conditions where initiation fails at other locations in the viral genome, or if replication of the viral genome is not complete. Replication initiation at DS may be required to complete DNA synthesis of the regions between the DS and FR, especially if these EBNA1 binding sites function as replication fork barriers. This arrangement of OriP elements may promote recombination structures associated with replication fork stalling, initiation, and temination events.

### Recombination structures form at OriP

Evidence that recombination-like structures formed at OriP were provided by one and two-dimensional agarose gel electrophoresis studies (Figs. 5 and 6). The formation of a vertical spike emerging from the 2n species in two-dimensional agarose gels has been interpreted as recombination-like structures, which include Holliday junctions and hemicatenanes [41], [42]. The recombination-like structures at OriP were detected after the appearance of the bubble arc in mid S phase and persisted through the remainder of late S and G2 (Fig. 5B, fraction 26–30). A similar cell cycle pattern of recombination structures forming after origin initiation has been observed in lower eukaryotes [40], [58]. Further evidence for recombination junctions at OriP was provided by 1-D agarose gel analysis (Fig. 6). Recombination junctions can be stabilized by CTAB extraction which inhibits branch migration [46]. We were able to show that CTAB stabilized a form of OriP DNA that migrated at higher molecular mass (∼2n) (Fig. 6A). The CTAB-stabilized species was enriched in S phase (Fig. 6B) and senstive to T7endoI (Fig. 6C). Furthermore, T7endoI eliminated the vertical spike in 2D gel analysis (Figs. S5 and S6), indicating that the CTAB stabilized structure in 1D gels corresponds to the vertical spike in the 2D gel analysis. The vertical spike in 2D gels was also sensitive to heat treatment at 65°C, suggesting that this structure is sensitive to branch migration (Fig. S6). Since T7endoI has known specificity for HJ structures [59], we consider it likely that HJ and related recombinational structures form at OriP.

### Implications for EBV episomal maintenance

**Figure 7:**
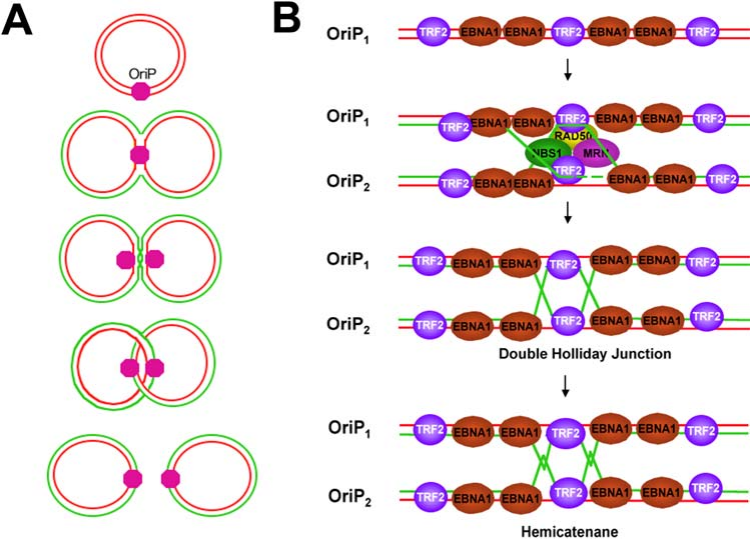
Model of recombination junctions formed at OriP and their function in sister-chromatid cohesion. A) Schematic of EBV episome DNA replication with fork pausing and terminating at OriP. Recombination structures are formed at OriP and provide sister chromatid cohesion through topological linkages. Resolution of these linkages in anaphase promotes genome segregation and stable plasmid maintenance. B) Speculative model showing MRN and recombination structures that may form at DS. These include strand invasion, double Holliday junctions, and hemicatenane of nascent strand DNA.

The prevailing model for episomal maintenance of gammaherpesviruses argues that replicated viral genomes will distribute stochastically to daughter cells through a non-specific tethering of newly replicated genomes to cellular chromosomes [60]–[62]. More recent studies have revealed that the viral episomes maintain a stable copy number through a mechanism involving sister chromatid cohesion and anaphase segregation, similar to cellular chromosomes [63], [64]. Our findings suggest that recombination-like junctions, which form at OriP, may contribute to the sister-chromatid cohesion required for genome segregation (Fig. 7A). Consistent with this model, we found that topologically linked dimers of EBV accumulate in late S and G2/M phase of the cell cycle (Fig. 6D). We propose that MRN components, which localize at OriP and contribute to OriP replication and maintenance function, are important for the establishment of these linked structures (Fig. 7B). Others have shown that MRN components contribute to HJ formation in mammalian cells [65]. MRN may work in conjunction with TRF2 at DS to stimulate strand invasion, in a manner similar to that proposed at telomere T-loops [34]. While the precise structure of the recombination-like junctions are not yet known, we suggest that these junctions contibute to the sister chromatid cohesion necessary for homologous DNA repair and for chromosome segregation [66]. Interestingly, the origin recognition complex (ORC) promotes sister chromatid cohesion in yeast (ORC) [67], and it is possible that OriP recruits ORC to generate cohesion between sister viral genomes. This suggests that the origin function of OriP is mechanistically linked to the plasmid maintenance function. These findings provide a new mechanistic paradigm for OriP plasmid maintenance, and have implications for the episome maintenance mechanisms of related viruses, including KSHV and HPV. These findings may also have implications for the mechanisms of generating sister chromatid cohesion at cellular chromosome sites, like telomere repeats and origins of replication, where recombinational junctions are likely to form.

## Methods

### Cell lines and antibodies

EBV positive lymphoblastoid cell lines (LCLs) and Burkitt lymphoma cell lines (Raji, MutuI, and Namalwa) were maintained in RPMI medium supplemented with 10% fetal bovine serum, glutamine, penicillin, and streptomycin sulfate (Cellgro). D98/HR1 (EBV^+^ adherent cells), Hela, and 293 (EBV-adherent cells) cells were maintained in DMEM medium supplemented with 10% fetal bovine serum, glutamine, penicillin, and streptomycin sulfate (Cellgro). Mre11, Nbs1 mutant, and reconstituted cells were provided by M.D. Weitzman [43]. Lymphoblastoid cell lines from NBS1 mutated individuals, GM15808 and GM07078 were purchased from Coriel Institute (Camden, NJ). The following rabbit polyclonal antibodies were used: anti-Mre11 (NB 100-142; Novus), anti-Nbs1 (Nb 100-277; Novus), anti-Brca1 (Abcam), and control rabbit immunoglobulin G (IgG; Jackson Laboratories). Rabbit polyclonal anti-EBNA1 and Trf2 were raised against a recombinant full-length EBNA1 and Trf2.

### Cell Cycle Synchronization

Cell cycle fractionation using centrifugal elutriation was performed with a modified Beckman JE 5.0 using counter flow rates for EBV positive B-lymphocytes as described previously[68], [69]. Alternatively, asynchronous Raji cells were arrested in G1/S using double thymidine method and released into S phase, then collected at different time points at early S (2 h), middle S (4 h), and late S (6 h), or at G2/M by treatment with 150 ng Nocodozole (Sigma) for 18 h [70].

### CTAB Isolation of DNA

DNA extraction with cetyltrimethylammonium bromide (CTAB) was essentially as described [46]. See supplemental methods for additional details.

### Two Dimensional Gel Electrophoresis

Two dimensional gel electrophoresis was performed essentially as described previously, except that DNA was prepared using the CTAB method [46], [71].

### Additional Methods

Chromatin Immunoprecipitation, FACS analysis of cell cycle, and DNA replication assays have been described previously[22]. Primers used for real time PCR analysis of ChIP assays were: OriP (DS) (atgtaaataaaaccgtgacaggctcat; ttacccaacgggaagcatatg), OriR (ggccacgctgataaagttgt ; ctagaaaccctggcgaccat) and OriLyt (cgtcttactgcccagcctact; agtgggagggcaggaaat). siRNA were synthesized as duplex RNA (Dharmacon Inc.) with the following target sequences for Luciferase control (cgtacgcggaatacttcga) and a pool of three target sequences for Mre11 (cctgcctcgagttattaag; ctgcgagtggactatagtg; gatgccattgaggaattag,) as described previously [72]. siRNA for NBS1 is commercially available as Smartpool products (Dharmacon Inc.). Plasmid or siRNA controls were used in parallel for each siRNA experiment. Pulse field electrophoresis of EBV episomes purified from agarose plugs were described previously [73].

## Supporting Information

Text S1 Supplementary Methods (0.04 MB DOC)

Figure S1 MRE11 and BRCA1 siRNA depletion does not cause cell cycle arrest. FACS analysis of propidium iodide stained cells after control or siRNA depletion of MRE11 (A), or NBS1 (B) used for replication assays shown in Figure 2.
(4.04 MB TIF)

Figure S2 Evidence for integrated EBV genomes in NBS1 GM15808. Total genomic DNA was isolated from Raji, Namalwa, NBS1 GM15808, NBS1 GM07078, LCL 3472, or LCLAW7, and linearized with BamHI. DNA was then analyzed by Southern blot and hybridized to probes specific for the left junction of the terminal repeats (TR_L) (panel A), the right junction of the terminal repeat (TR_R) (panel B), or OriP region (panel C). Different fragments size of the terminal repeats is indicative of integrated forms.
(1.88 MB TIF)

Figure S3 Early S phase recruitment of MRE11, NBS1, and BRCA1 to DS. A) Chromatin immunoprecipitation of EBV positive Raji cells was analyzed at different times in the cell cycle with antibodies to MRE11, NBS1, BRCA1 and control IgG. Immunoprecipitated DNA was quantified by real-time PCR with primers specific for DS or control OriLyt (OL) regions of the EBV genome. ChIP values were presented as percentage of input DNA. B) ChIP assays were used to measure MRE11, NBS1, and BRCA1 binding to OriP in asynchronous Raji cells (Asy) or Raji cells treated with 5 mM HU for 1 hr (HU), or 9 Gy of gamma irradiation (Gy). C) FACS profile of propidium iodide treated Raji cells synchronized by double thymidine block and used for ChIP assays shown in panel A.
(3.98 MB TIF)

Figure S4 Formation of X-structures at OriP in Raji cells. Two-dimensional neutral agarose gels were used to analyze DNA extracted from Raji cells after centrifugal elutriation. The cell cycle fractions are indicated in the above right, and correspond to nearly identical cell cycle distribution as shown for MutuI cells (Figure 4A). Raji cell DNA was extracted by CTAB method, digested with PvuII and visualized by Southern blot hybridization to an OriP specific probe.
(7.13 MB TIF)

Figure S5 T7EndoI Sensitivity of Recombinational Structures at OriP. MutuI DNA was extracted by CTAB, linearized with PvuII and then further treated with a) mock (Control), b) mung bean, or c) T7EndoI nuclease. DNA was then analyzed by 2-D neutral agarose gel electrophoresis and Southern blot probed for OriP. PhosphorImager gels of two independent experiments are above (Expt I and Expt II), and a schematic interpretation is shown below.
(15.91 MB TIF)

Figure S6 Thermal Sensitivity of Recombinational Structures at OriP. Mutu I DNA was extracted with CTAB, linearized with PvuII, and then treated with either T7 EndoI nuclease, 65°C for 30 min, or mock treatment (control). DNA was analyzed by 2D gel and Southern blotting with OriP-specific probe. B) A schematic interpretation of the salient DNA structures is shown.
(8.90 MB TIF)
