# Supplementary material for: A Role for MRE11, NBS1, and Recombination Junctions in Replication and Stable Maintenance of EBV Episomes
Source: PLoS One. 2007 Dec 5;2(12):e1257. doi: 10.1371/journal.pone.0001257 (PMC2094660; doi:10.1371/journal.pone.0001257)
Supplement: Text S1 — (0.04 MB DOC) [file pone.0001257.s001.doc]

Supplemental Methods

**CTAB Isolation of DNA**

DNA extraction with cetyltrimethylammonium bromide (CTAB) was essentially as described [46]. Briefly, nuclei were isolated by resuspending cells in RSB buffer (10 mM Tris-HCl pH8, 10 mM NaCl, 3mM MgCl_2_) at a concentration of 2.5x10^7^ cells/ml and incubating the sample in ice for 5 min. An equal volume of 0.2% NP-40 in RSB buffer was added followed by incubation in ice for additional 10 min. The nuclei so obtained were pelleted by centrifugation. Sequentially, 2 ml of water, 500 μl of RNase A (10 mg/ml), 500 μl of proteinase K (20 mg/ml), and 2.5 ml of solution I (2% cetyltrimethylammonium bromide [CTAB], 1.4 M NaCl, 100 mM Tris [pH 7.6], 25 mM EDTA, 20 mM HCC) were added to the Nuclei, and the sample was incubated at 37°C for 30 min. The proteins were extracted with 3 ml of chloroform-isoamyl alcohol (24:1), and the supernatant was transferred to a new tube. DNA was precipitated by adding 2 volumes of solution II (1% CTAB, 50 mM Tris [pH 7.6], 10 mM EDTA, 5 mM HCC) and centrifugation for 5 min at full 10000 rpm. The DNA was resuspended in 0.5 ml of solution III (1.4 M NaCl, 10 mM Tris [pH 7.6], 1 mM EDTA, 1 mM HCC), precipitated with 1 volume of isopropanol, and centrifuged as above. The pellet was briefly rinsed with 70% ethanol, air dried, resuspended in TMS buffer ( 10 mM Tris-HCl pH 7.6, 2 mM MgCl2, 50 µM Spermidine ), and then subject to restriction digest and Southern blot analysis.

**Two-Dimensional Neutral Agarose Gel Analysis**

DNA (10 μg) from different phases of cell cycle was digested in 1X NEB buffer 2 (New England Biolabs) with PvuII (50 units) for 3 hr at 37°C. After digestion, DNA was precipitated by adding 2 vol of ethanol and 0.1 vol of 3 M sodium acetate. DNA pellet was dissolved in 30 ul of loading buffer (10 mM Tris-HCl (pH 7.5), 3 mM MgCl_2_, 1 mM EDTA (pH 8), 15% Ficoll 400, 0.25% bromophenol blue), and samples were kept on ice until electrophoresis.

Neutral/neutral 2D electrophoresis was performed as described (ref) with the following modifications (details available upon request). First dimension gel: 0.4% Seakem Gold agarose (FMC) in TBE+MgCl_2_ (89 mM Tris-borate [pH 8.3], 1 mM EDTA [pH 8], 3 mM MgCl_2_), cast in preformed 0.5 cm wide lanes, run for 20 hr at 1 V/cm, room temperature. Lanes were stained for 0.5 hr in TBE+MgCl_2_ plus 0.3 μg/ml ethidium bromide (TBE+MgCl_2_+EtBr) and cast into a 0.9% Seakem GTG agarose (FMC) gel in TBE+ MgCl_2_+EtBr. Second dimension: 8.5 hr at 6 V/cm, 4°C (buffer temperature did not exceed 15°C). The gel was soaked in 5 volumes 0.25 N HCl for 12 min before blotting.

Restriction enzyme, T7 endonuclease I, and mung bean nuclease digestion. Restriction enzyme, T7 endonuclease I, and mung bean nuclease digests were carried out in the conditions recommended by the supplier (New England Biolabs). Briefly, DNA (2-3 μg) was digested with Ecor1/Kpn1 or Ear1 (10 units) at 37 °C for 2h, and the incubation was further continued for another 15 min in the presence of T7 endonuclease I or mung bean nuclease. The samples were separated on agarose gel electrophoresis and analyzed by southern blot.

For the plasmid experiments, Nbs wt or mutant cell were nucleofected with oriP plasmid and after 3 days of post transfection, DNA was isolated by CTAB and restriction digestion was done as above.
