# Supplementary figures and images for: A Role for MRE11, NBS1, and Recombination Junctions in Replication and Stable Maintenance of EBV Episomes
Source: PLoS One. 2007 Dec 5;2(12):e1257. doi: 10.1371/journal.pone.0001257 (PMC2094660; doi:10.1371/journal.pone.0001257)

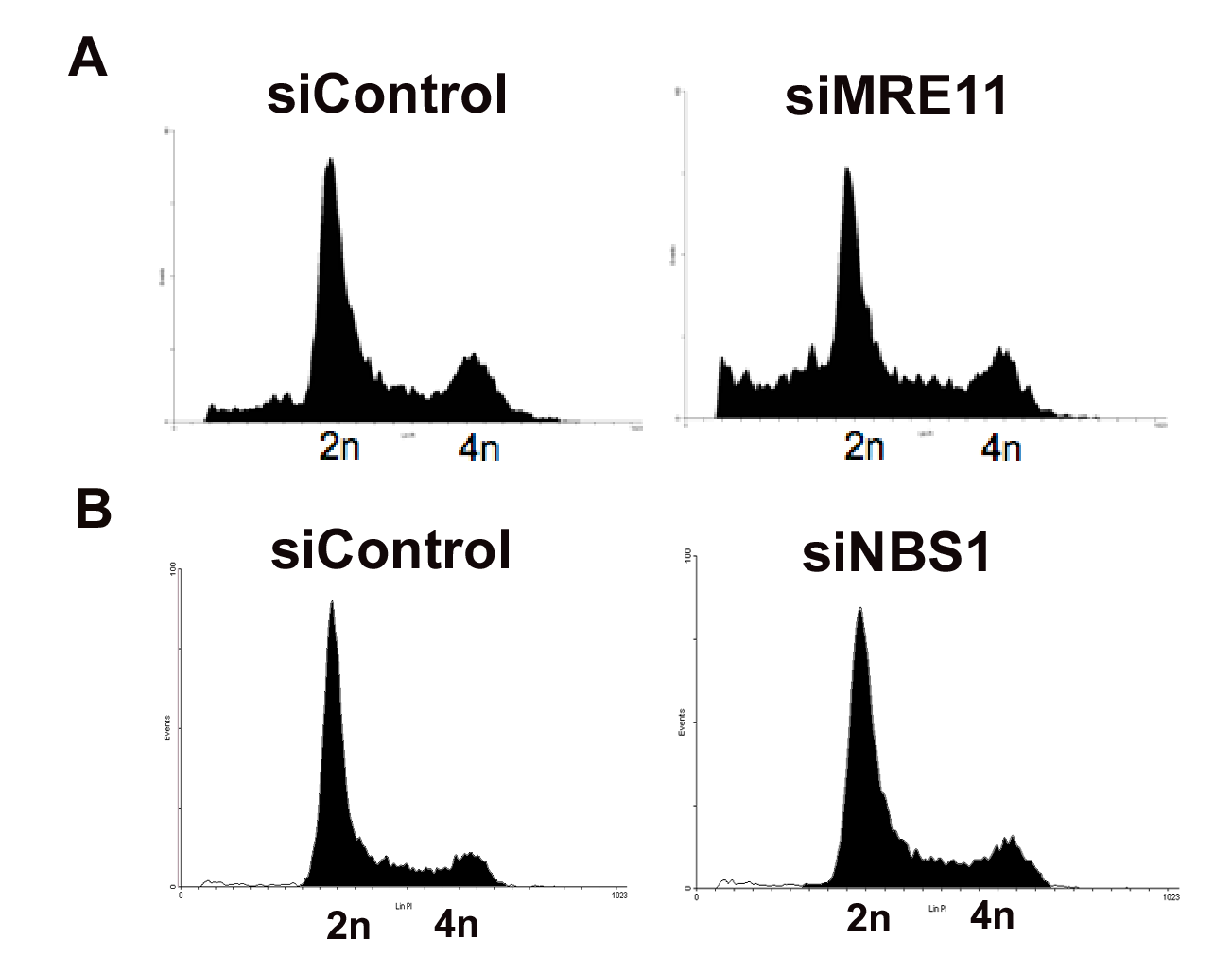

Supplement: Figure S1 — MRE11 and BRCA1 siRNA depletion does not cause cell cycle arrest. FACS analysis of propidium iodide stained cells after control or siRNA depletion of MRE11 (A), or NBS1 (B) used for replication assays shown in Figure 2. (4.04 MB TIF) [file pone.0001257.s002.tif]

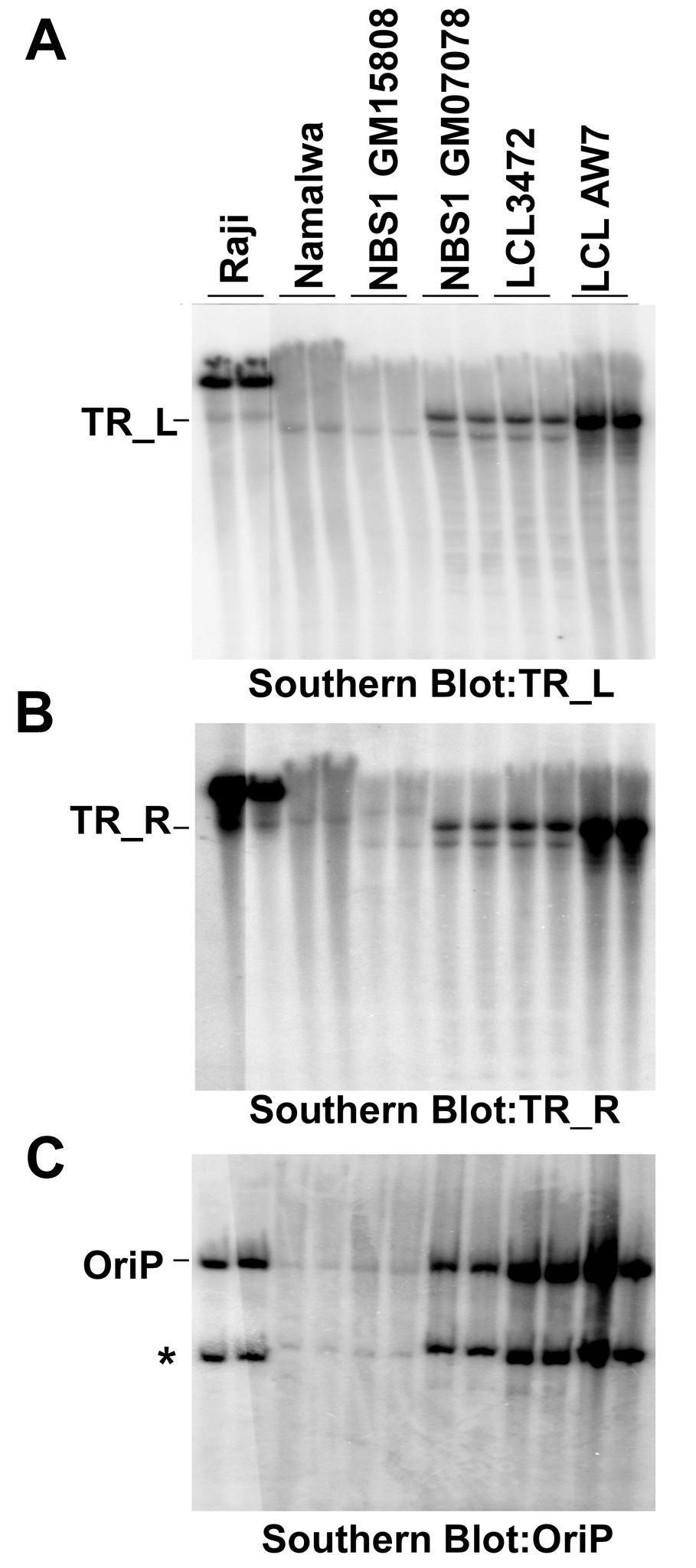

Supplement: Figure S2 — Evidence for integrated EBV genomes in NBS1 GM15808. Total genomic DNA was isolated from Raji, Namalwa, NBS1 GM15808, NBS1 GM07078, LCL 3472, or LCLAW7, and linearized with BamHI. DNA was then analyzed by Southern blot and hybridized to probes specific for the left junction of the terminal repeats (TR_L) (panel A), the right junction of the terminal repeat (TR_R) (panel B), or OriP region (panel C). Different fragments size of the terminal repeats is indicative of integrated forms. (1.88 MB TIF) [file pone.0001257.s003.tif]

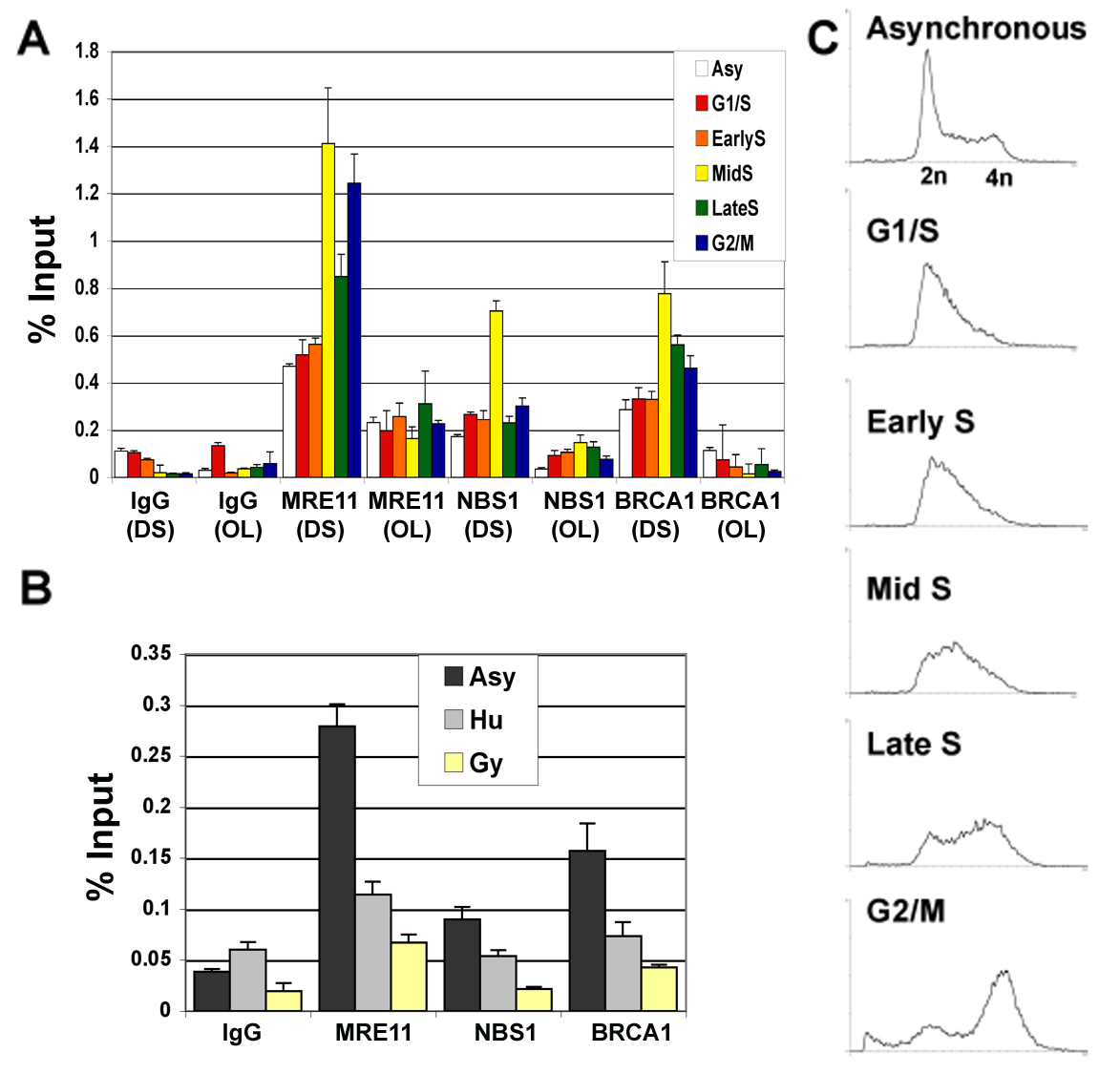

Supplement: Figure S3 — Early S phase recruitment of MRE11, NBS1, and BRCA1 to DS. A) Chromatin immunoprecipitation of EBV positive Raji cells was analyzed at different times in the cell cycle with antibodies to MRE11, NBS1, BRCA1 and control IgG. Immunoprecipitated DNA was quantified by real-time PCR with primers specific for DS or control OriLyt (OL) regions of the EBV genome. ChIP values were presented as percentage of input DNA. B) ChIP assays were used to measure MRE11, NBS1, and BRCA1 binding to OriP in asynchronous Raji cells (Asy) or Raji cells treated with 5 mM HU for 1 hr (HU), or 9 Gy of gamma irradiation (Gy). C) FACS profile of propidium iodide treated Raji cells synchronized by double thymidine block and used for ChIP assays shown in panel A. (3.98 MB TIF) [file pone.0001257.s004.tif]

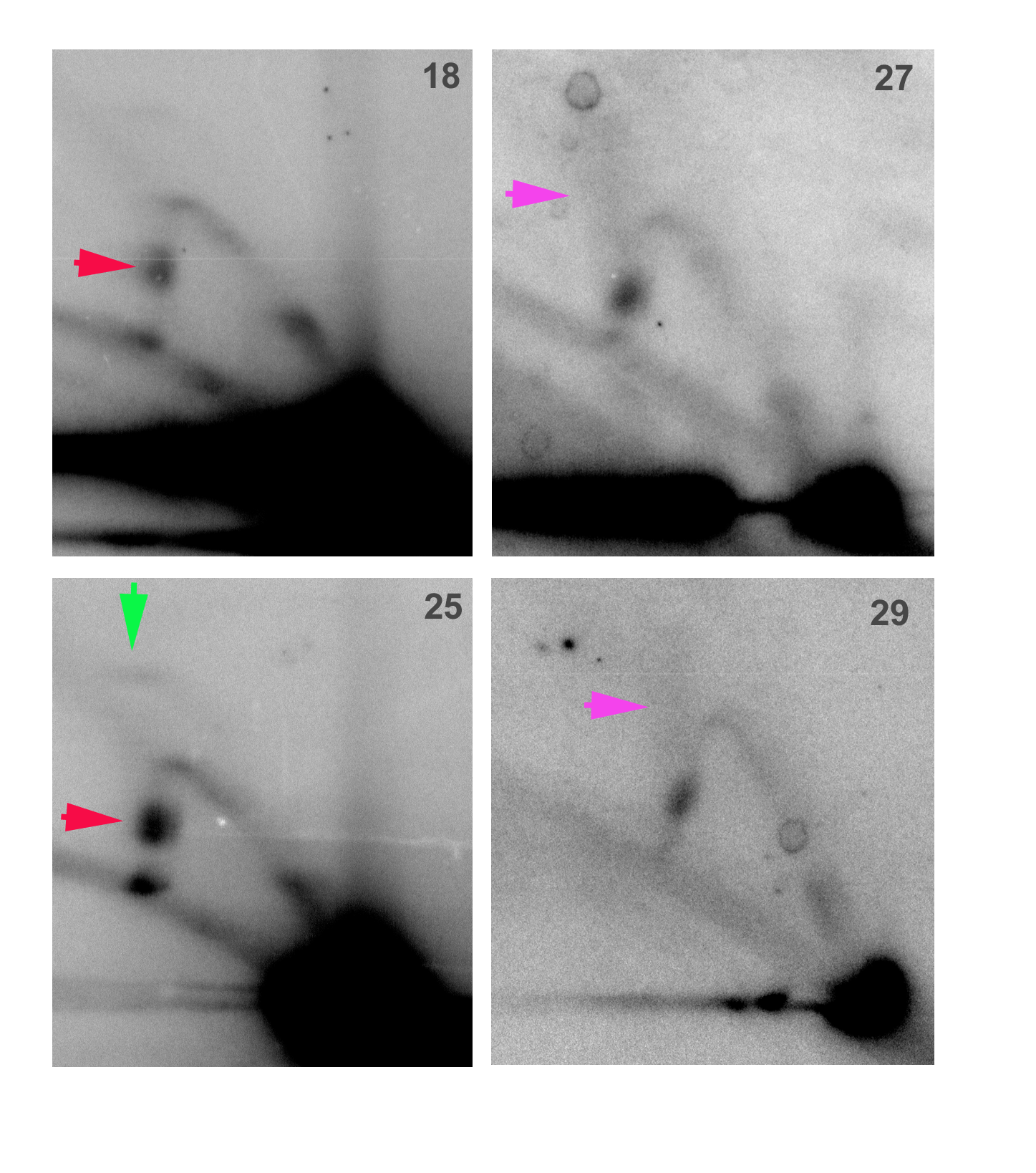

Supplement: Figure S4 — Formation of X-structures at OriP in Raji cells. Two-dimensional neutral agarose gels were used to analyze DNA extracted from Raji cells after centrifugal elutriation. The cell cycle fractions are indicated in the above right, and correspond to nearly identical cell cycle distribution as shown for MutuI cells (Figure 4A). Raji cell DNA was extracted by CTAB method, digested with PvuII and visualized by Southern blot hybridization to an OriP specific probe. (7.13 MB TIF) [file pone.0001257.s005.tif]

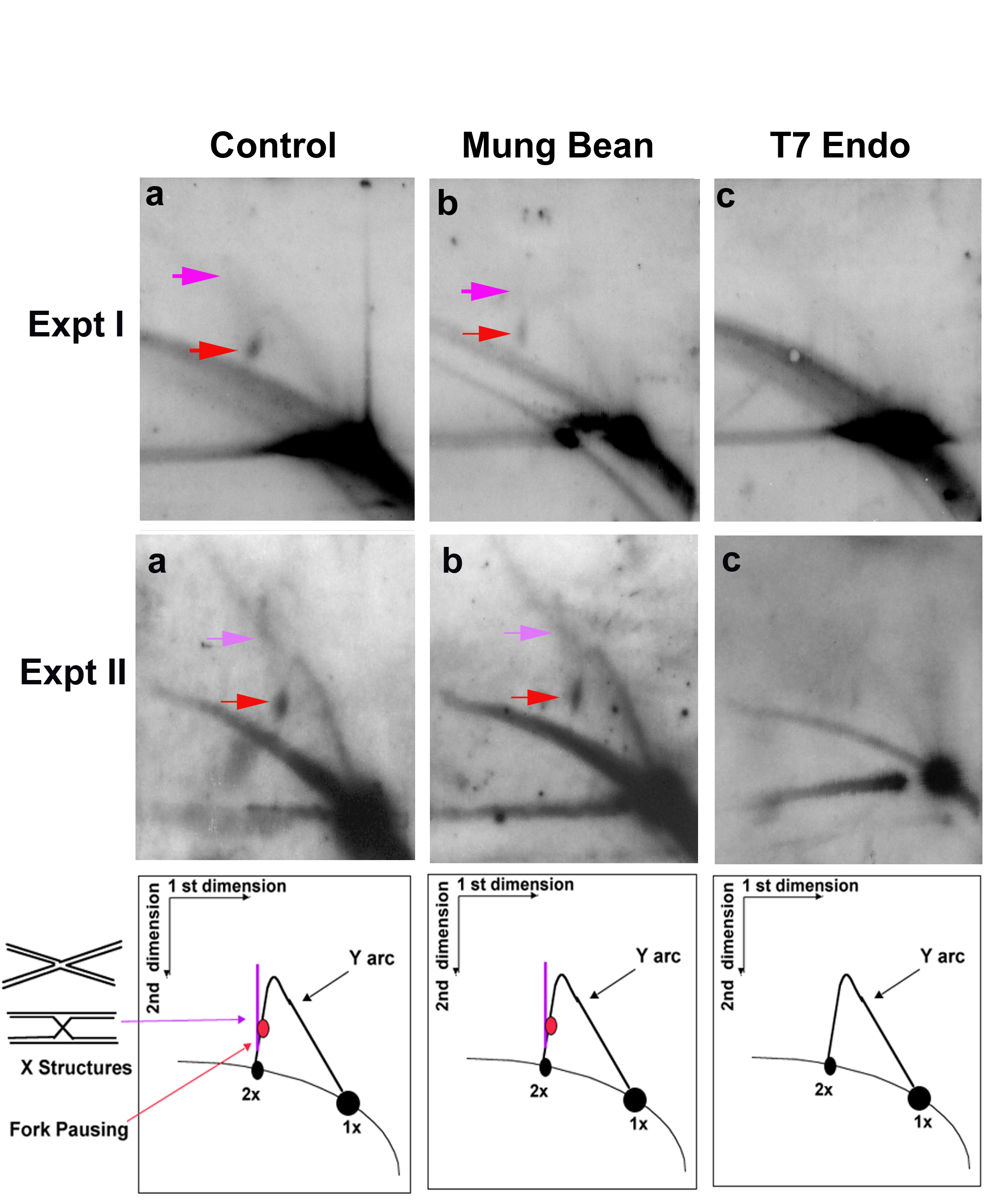

Supplement: Figure S5 — T7EndoI Sensitivity of Recombinational Structures at OriP. MutuI DNA was extracted by CTAB, linearized with PvuII and then further treated with a) mock (Control), b) mung bean, or c) T7EndoI nuclease. DNA was then analyzed by 2-D neutral agarose gel electrophoresis and Southern blot probed for OriP. PhosphorImager gels of two independent experiments are above (Expt I and Expt II), and a schematic interpretation is shown below. (15.91 MB TIF) [file pone.0001257.s006.tif]

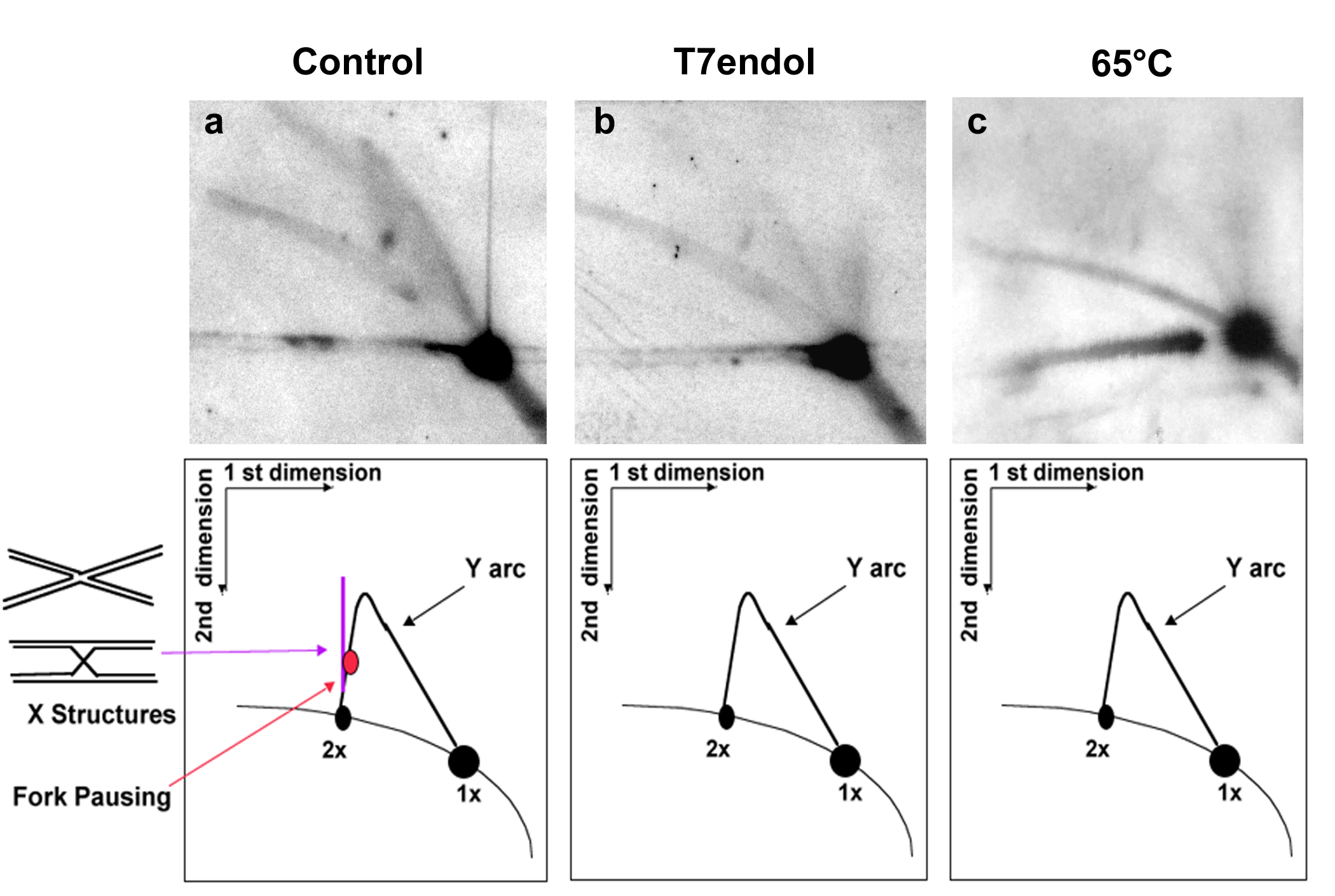

Supplement: Figure S6 — Thermal Sensitivity of Recombinational Structures at OriP. Mutu I DNA was extracted with CTAB, linearized with PvuII, and then treated with either T7 EndoI nuclease, 65°C for 30 min, or mock treatment (control). DNA was analyzed by 2D gel and Southern blotting with OriP-specific probe. B) A schematic interpretation of the salient DNA structures is shown. (8.90 MB TIF) [file pone.0001257.s007.tif]
